
JOURNAL INFORMATION
==============================
NLM Title Abbreviation: ScientificWorldJournal
Journal ID: ScientificWorldJournal
NLM Journal ID: 101131163
Journal ID: TSWJ

The Scientific World Journal

ISSN: 2356-6140
EISSN: 1537-744X
Publisher: Wiley

ARTICLE INFORMATION
==============================
PMCID: PMC5901351
PMID: 18167615
DOI: 10.1100/tsw.2007.312
Article ID: 303689
Article version: 1
Subjects: Directions in Science

Met Receptor Subcellular Localization Depends on E-Cadherin Function

Reshetnikova Galina *
Institute of Cytology, Russian Academy of Sciences, St. Petersburg, Russia
*Galina Reshetnikova: greshet@mail.cytspb.rssi.ruAcademic Editor: Martin Goette

Publication date: 2007
Electronic publication date: 2007 Dec 18
Volume: 7
First page: 2009
Last page: 2011
Received 2007 Jul 13; Revised 2007 Nov 29; Accepted 2007 Dec 4
Copyright: Copyright © 2007 Galina Reshetnikova.
Copyright year: 2007
License: This is an open access article distributed under the Creative Commons Attribution License, which permits unrestricted use, distribution, and reproduction in any medium, provided the original work is properly cited.
License URL: https://creativecommons.org/licenses/by/3.0/

Keywords: E-cadherin, hepatocyte growth factor, Met, signaling, epithelial cell polarization

Funding: Citrus Research & Development Foundation Russian Foundation for Basic Research

==============================
Directions in Science
*TheScientificWorldJOURNAL* (2007) 7, 2009–2011
ISSN 1537-744X; DOI 10.1100/tsw.2007.312

TheScientificWorldJOURNAL
www.thescientificworld.com

### Met Receptor Subcellular Localization Depends on E-Cadherin Function

Galina Reshetnikova
*Institute of Cytology, Russian Academy of Sciences, St. Petersburg*

**E-mail**: greshet@mail.cytspb.rssi.ru

*Received July 13, 2007; Revised November 29, 2007; Accepted December 4, 2007; Published December 18, 2007*

**KEYWORDS:** E-cadherin, hepatocyte growth factor, Met, signaling, epithelial cell polarization

Overexpression of the HGF (hepatocyte growth factor) receptor Met is correlated with metastasis and poor outcome in cancer. Upon HGF binding, Met mediates multiple cellular processes, including mitogenesis, motility, and morphogenesis[1].

Met and HGF likely play a key role in regulating many aspects of embryonic development, including epithelial-mesenchymal transition[2], migration of muscle cells[3], and kidney and mammary gland formation[4,5]. Met signaling also facilitates wound healing and tissue regeneration[6]. Met oncogenic activation was shown to play a significant role in promoting tumor cell invasion and metastasis[7]. *In vitro*, activation of Met by HGF induces disassembly of the cell-cell contacts, followed by cell migration[8]. E-cadherin is the central protein of adhesion complexes in epithelial cells. E-cadherin is a transmembrane protein whose cytoplasmic domains interact with catenins to connect to the cytoskeleton[9]. However, the mechanism involved in down-regulation of E-cadherin function by Met is poorly understood. One important question that has not been resolved is whether E-cadherin and Met have physically interacted at the plasma membrane.

There are only a few data suggesting that Met might tightly interact with the E-cadherin–dependent adhesion complex. Met has been shown to be selectively localized at the basolateral area of polarized epithelial cells *in vivo*[10]. Also, Met and E-cadherin were found colocalized on the membrane of human carcinoma cells *in vitro*[11,12]. Moreover, in MDCK cells, both proteins were colocalized at the cell-cell adhesion site and underwent coendocytosis upon HGF stimulation[13]. We have now shown that Met is tightly associated with E-cadherin at the site of cell-cell contacts[14]. It appears that the interaction of Met and E-cadherin occurs by direct binding in their extracellular domains as part of the process of maturation of adhesive junctions. The cadherin-mediated cell adhesion divided the plasma membrane into apical and basolateral domains that have different protein compositions[15]. Recently, new data concerning the dynamics of plasma membrane proteins in epithelial cells were published. It was demonstrated that proteins that are normally localized at the plasma membrane in polarized epithelial cells distributed to intacellular compartments when cell polarity is lost or not yet established because of redirection of plasma membrane transport pathways into intracellular vesicles[16,17]. Interestingly, such membrane markers like E-cadherin, β-catenin, occludin, JAM-1, Na/K-ATPase, and syntaxins were accumulated in the unique intracellular storage compartment upon destruction of cell-cell adhesion[18]. Our work demonstrated that E-cadherin–negative BT-549 cells delivered Met to an intracellular location. However, forced expression of E-cadherin restored cell-cell contacts and recruited Met to the membrane.

©2007 with author.
Published by TheScientificWorld; www.thescientificworld.com

On the basis of these observations, we speculate that Met sorted to the basolateral domain in polarized cells.

Previous reports also showed that E-cadherin formed ligand-independent complexes with epidermal growth factor receptor (EGFR); this interaction required the extracellular domain of E-cadherin[19,20]. Other classical cadherins, N-cadherin and VE-cadherin, are also implicated in regulation of receptor tyrosine kinases (RTKs), FGFR-1, and VEGFR2, respectively[21,22]. Phosphatases have also been localized to the adhesive complex and, in some cases, have been shown to interact with cadherins[22]. Furthermore, DEP-1 and LAR protein-tyrosine phosphatase play a role in inactivation of Met through their physical interaction, which specifically occurs upon confluent condition[23,24]. It may be that the RTK-cadherin interaction allows tightly regulated maintenance of the dephosphorylated state of receptors until the ligand is present. In addition, there are publications documenting that, in polarized epithelial cells, the signaling pathways initiated by EGFR differ at the apical and basolateral cell membrane domain[25,26] Taken together, these results indicate a dynamic relationship between RTKs and E-cadherin that regulates the function of both molecules.

Our studies provided some insight into the mechanism of Met and E-cadherin interaction; we have shown that these proteins could interact extracellularly on the cell membrane[14]. To better understand the relationship between E-cadherin and Met, we assessed Met localization in cells that form mature E-cadherin–dependent adhesion HT-29 and cells that have lost E-cadherin expression BT-549. Our data show that Met and E-cadherin colocalize at the site of cell-cell contacts and are cross-linkable by their extracellular domains in polarized HT-29 cells. In E-cadherin–negative BT-549 cells, Met is localized in the cytoplasm. However, upon transfection of BT-549 cells with cadherin, Met is recruited to the membrane. We suggested that the direct external interaction is important in presentation of Met or stabilization of Met on the membrane. Furthermore, its proximity to E-cadherin may be critical for proper localization such that activation can trigger events that result in dissolution of adhesion.

An increasing amount of experimental evidences imply a higher-order level of regulation of RTK signaling in polarized epithelial cells based on the spatial compartmentalization of receptors. Bussolino et al. proposed the concept “of dynamic signaling membrane modules…where different molecules interact to make a function possible”[27]. It seems, that E-cadherin–dependent adhesion sites may serve as a micromodule for the accumulation of different signaling proteins, such as RTKs, β-catenin, phosphotase, and others. If this is true, further studies are required to establish how the formation and dissolution of the “signaling module” is regulated.

### ACKNOWLEDGMENTS

This work was supported by a grant from CRDF-RFBR (RUB1-2868-ST-07/07-04-91154).

### REFERENCES

1. Zhang, Y.W. and Vande Woude, G.F. (2003) HGF/SF-met signaling in the control of branching morphogenesis and invasion. *J. Cell. Biochem*. **88,** 408–417.
2. Thiery, J.P. (2002) Epithelial-mesenchymal transitions in tumour progression. *Nat. Rev. Cancer* **2,** 442–454.
3. Bladt, F., Riethmacher, D., Isenmann, S., Aguzzi, A., and Birchmeier, C. (1995) Essential role for the c-met receptor in the migration of myogenic precursor cells into the limb bud. *Nature* **376,** 768–771.
4. Santos, O.F., Barros, E.J., Yang, X.M., Matsumoto, K., Nakamura, T., Park, M., and Nigam, S.K. (1994) Involvement of hepatocyte growth factor in kidney development. *Dev. Biol*. **163,** 525–529.
5. Yang, Y., Spitzer, E., Meyer, D., Sachs, M., Niemann, C., Hartmann, G., Weidner, K.M., Birchmeier, C., and Birchmeier, W. (1995) Sequential requirement of hepatocyte growth factor and neuregulin in the morphogenesis and differentiation of the mammary gland. *J. Cell Biol*. **131,** 215–226.
6. Nusrat, A., Parkos, C.A., Bacarra, A.E., Godowski, P.J., Delp-Archer, C., Rosen, E.M., and Madara, J.L. (1994) Hepatocyte growth factor/scatter factor effects on epithelia. Regulation of intercellular junctions in transformed and nontransformed cell lines, basolateral polarization of c-met receptor in transformed and natural intestinal epithelia, and induction of rapid wound repair in a transformed model epithelium. *J. Clin. Invest*. **93,** 2056–2065.

7. Boccaccio, C. and Comoglio, P.M. (2006) Invasive growth: a MET-driven genetic programme for cancer and stem cells. *Nat. Rev. Cancer* **6,** 637–645.
8. Rosario, M. and Birchmeier, W. (2003) How to make tubes: signaling by the Met receptor tyrosine kinase. *Trends Cell Biol*. **13,** 328–335.
9. Gumbiner, B.M. (2000) Regulation of cadherin adhesive activity. *J. Cell Biol*. **148,** 399–404.
10. Crepaldi, T., Pollack, A.L., Prat, M., Zborek, A., Mostov, K., and Comoglio, P.M. (1994) Targeting of the SF/HGF receptor to the basolateral domain of polarized epithelial cells. *J. Cell Biol*. **125**, 313–320.
11. Matteucci, E., Ridolfi, E., and Desiderio, M.A. (2006) Hepatocyte growth factor differently influences Met-E-cadherin phosphorylation and downstream signaling pathway in two models of breast cells. *Cell. Mol. Life Sci*. **63,** 2016–2026.
12. Gotte, M., Kersting, C., Radke, I., Kiesel, L., and Wulfing, P. (2007) An expression signature of syndecan-1 (CD138), E-cadherin and c-met is associated with factors of angiogenesis and lymphangiogenesis in ductal breast carcinoma in situ. *Breast Cancer Res*. **9,** R8.
13. Kamei, T., Matozaki, T., Sakisaka, T., Kodama, A., Yokoyama, S., Peng, Y.F., Nakano, K., Takaishi, K., and Takai, Y. (1999) Coendocytosis of cadherin and c-Met coupled to disruption of cell-cell adhesion in MDCK cells--regulation by Rho, Rac and Rab small G proteins. *Oncogene* **18,** 6776–6784.
14. Reshetnikova, G., Troyanovsky, S., and Rimm, D.L. (2007) Definition of a direct extracellular interaction between Met and E-cadherin. *Cell Biol. Int*. **31,** 366–373.
15. Drubin, D.G. and Nelson, W.J. (1996) Origins of cell polarity. *Cell* **84,** 335–344.
16. Low, S.H., Miura, M., Roche, P.A., Valdez, A.C., Mostov, K.E., and Weimbs, T. (2000) Intracellular redirection of plasma membrane trafficking after loss of epithelial cell polarity. *Mol. Biol. Cell* **11,** 3045–3060.
17. Tuma, P.L., Nyasae, L.K., and Hubbard, A.L. (2002) Nonpolarized cells selectively sort apical proteins from cell surface to a novel compartment, but lack apical retention mechanisms. *Mol. Biol. Cell* **13,** 3400–3415.
18. Ivanov, A.I., Nusrat, A., and Parkos, C.A. (2004) Endocytosis of epithelial apical junctional proteins by a clathrin-mediated pathway into a unique storage compartment. *Mol. Biol. Cell* **15,** 176–188.
19. Fedor-Chaiken, M., Hein, P.W., Stewart, J.C., Brackenbury, R., and Kinch, M.S. (2003) E-cadherin binding modulates EGF receptor activation. *Cell Commun. Adhes*. **10,** 105–118.
20. Qian, X., Karpova, T., Sheppard, A.M., McNally, J., and Lowy, D.R. (2004) E-cadherin-mediated adhesion inhibits ligand-dependent activation of diverse receptor tyrosine kinases. *EMBO J*. **23,** 1739–1748.
21. Suyama, K., Shapiro, I., Guttman, M., and Hazan, R.B. (2002) A signaling pathway leading to metastasis is controlled by N-cadherin and the FGF receptor. *Cancer Cell* **2,** 301–314.
22. Grazia Lampugnani, M., Zanetti, A., Corada, M., Takahashi, T., Balconi, G., Breviario, F., Orsenigo, F., Cattelino, A., Kemler, R., Daniel, T.O., and Dejana, E. (2003) Contact inhibition of VEGF-induced proliferation requires vascular endothelial cadherin, beta-catenin, and the phosphatase DEP-1/CD148. *J. Cell Biol*. **161,** 793–804.
23. Palka, H.L., Park, M., and Tonks, N.K. (2003) Hepatocyte growth factor receptor tyrosine kinase met is a substrate of the receptor protein-tyrosine phosphatase DEP-1. *J. Biol. Chem*. **278,** 5728–5735.
24. Machide, M., Hashigasako, A., Matsumoto, K., and Nakamura, T. (2006) Contact inhibition of hepatocyte growth regulated by functional association of the c-Met/hepatocyte growth factor receptor and LAR protein-tyrosine phosphatase. *J. Biol. Chem*. **281,** 8765–8772.
25. Amsler, K. and Kuwada, S.K. (1999) Membrane receptor location defines receptor interaction with signaling proteins in a polarized epithelium. *Am. J. Physiol*. **276,** C91–C101.
26. Bishop, W.P. and Wen, J.T. (1994) Regulation of Caco-2 cell proliferation by basolateral membrane epidermal growth factor receptors. *Am. J. Physiol*. **267,** G892–900.
27. Bussolino, F., Serini, G., Mitola, S., Bazzoni, G., and Dejana, E. (2001) Dynamic modules and heterogeneity of function: a lesson from tyrosine kinase receptors in endothelial cells. *EMBO Rep*. **2,** 763–767.

**This article should be cited as follows:**

Reshetnikova, G. (2007) Met receptor subcellular localization depends on E-cadherin function. *TheScientificWorldJOURNAL* **7**, 2009–2011. DOI 10.1100/tsw.2007.312.
